# Multimodal investigations of trans-endothelial cell trafficking under condition of disrupted blood-brain barrier integrity

**DOI:** 10.1186/1471-2202-11-34

**Published:** 2010-03-09

**Authors:** Nicola Marchi, Qingshan Teng, Minh T Nguyen, Linda Franic, Nirav K Desai, Thomas Masaryk, Peter Rasmussen, Silvia Trasciatti, Damir Janigro

**Affiliations:** 1 Department of Cerebrovascular Research Cell Biology, Cleveland Clinic Lerner College of Medicine, 9500 Euclid Ave, Cleveland, 44106, Ohio, USA; 2 Departments of Neurosurgery, Cleveland Clinic Lerner College of Medicine, 9500 Euclid Ave, Cleveland, 44106, Ohio, USA; 3 Neuroradiology, Cleveland Clinic Lerner College of Medicine, 9500 Euclid Ave, Cleveland, 44106, Ohio, USA; 4 AbiogenPharma Spa, Pisa, 57100, Italy

## Abstract

**Background:**

Stem cells or immune cells targeting the central nervous system (CNS) bear significant promises for patients affected by CNS disorders. Brain or spinal cord delivery of therapeutic cells is limited by the blood-brain barrier (BBB) which remains one of the recognized rate-limiting steps. Osmotic BBB disruption (BBBD) has been shown to improve small molecule chemotherapy for brain tumors, but successful delivery of cells in conjunction with BBBD has never been reported.

We have used a clinically relevant model (pig) of BBBD to attempt brain delivery of TALL-104, a human leukemic T cell line. TALL-104 cells are potent tumor killers and have demonstrated potential for systemic tumor therapy. The pig model used is analogous to the clinical BBBD procedure. Cells were injected in the carotid artery after labeling with the MRI T1 contrast agent GdHPDO3A. Contrast CT scans were used to quantify BBBD and MRI was used to detect Gd^++^-loaded cells in the brain. Transcranial Doppler was used to monitor cerebral blood flow. EEG recordings were used to detect seizures. Immunocytochemical detection (Cresyl Violet, anti-human CD8 for TALL-104, Evans Blue for BBB damage, GFAP and NEUN) was performed.

**Results:**

At the concentration used TALL-104 cells were tolerated. Incomplete BBBD did not allow cell entry into the brain. MRI scans at 24 and 48 hours post-injection allowed visualization of topographically segregated cells in the hemisphere that underwent successful BBBD. Perivascular location of TALL-104 was confirmed in the BBBD hemisphere by Cresyl violet and CD8 immunocytochemistry. No significant alteration in CBF or EEG activity was recorded during cell injections.

**Conclusions:**

Our data show that targeted CNS cell therapy requires blood-brain barrier disruption. MRI-detectable cytotoxic anti-neoplastic cells can be forced to transverse the BBB and accumulate in the perivascular space. The virtual absence of toxicity, the high anti-tumor activity of TALL-104, and the clinical feasibility of human osmotic BBBD suggest that this approach may be adopted to treat brain or spinal cord tumors. In addition, BBBD may favor CNS entry of other cells that normally lack CNS tropism.

## Background

The brain is protected by physical and vascular barriers, namely the skull and the blood-brain barrier (BBB). The system of capillaries forming the human BBB has approximately 20 m^2 ^of exchange surface with the brain parenchyma, and is situated a few microns from neurons and glial cells. In particular, the BBB controls the exchange of nutrients, xenobiotics and serum-derived factors between the systemic circulation and the brain, thus contributing to brain homeostasis necessary for the correct function of neurons [1]. At the cellular level, the BBB is composed of endothelial cells and glia. Endothelial cells are characterized by the presence of tight junctions, minimal pinocytic vesicles, and lack of fenestrations.

The restrictive nature of the BBB prevents significant penetration of many molecules and cells into the brain. As a result, while protecting the brain from harmful compounds, the BBB impedes or reduces access of therapeutic molecules to the brain [2]. This restriction is an important element contributing to our persistent inability to treat many CNS diseases, spanning from epilepsy to primary or metastatic brain tumors. The efficacy of new or future molecular approaches or exploiting of engineered cells, are and will be limited by BBB penetration. Stem cells or immune cells targeting the central nervous system bear significant promises for patients affected by CNS disorders. Brain or spinal cord delivery of therapeutic cells depends on a number of factors, including endothelial adhesion molecules, disruption of tight junctions, and penetration across the basal lamina surrounding the vessels [3]. Evidence suggests that under normal conditions cell entry into the brain occurs across larger vessels and venules [4-6]. However, to exert therapeutic actions it is desirable to gain access to the neuropil located in the brain parenchyma. Therefore, the usual pathway for immune trafficking has to be extended to the BBB proper, e.g., capillaries surrounded by astrocytic endfeet and pericytes.

Osmotic BBB disruption (BBBD) has been shown to improve small molecule chemotherapy for brain tumors [7] while its efficacy in promoting cell entry into the brain is still unclear [8]. The BBBD procedure leads to hemispheric disruption of the cerebrovasculature and has been clinically demonstrated to enhance the delivery of methotrexate to the brain [9] with tolerable side effects [7]. While chemotherapy after BBBD has already reached the clinical stage and demonstrated its therapeutic utility, animal models of BBBD are still viable tools for further advancements of chemo- and cell therapy. In particular, the pig model of BBBD is a faithful replica of the clinical reality, including the imitation of side effects [7,10,11].

Advances in our understanding of the cell and molecular biology of neurological diseases have been made in recent years. These advances have lead to the formulation of novel therapeutic means including cytotoxic cell therapy, such as lymphokine activated killer (LAK) cell, tumor infiltrating lymphocytes (TIL) and the more recent TALL-104 cells [12,13]. These therapies represent a promising approach [12,14], but share a common theme of low permeability through the BBB, impeding the access to brain targets or tumor cells; in fact, successful tumor invasion by these cells was only achieved by direct CNS injection [13,15]. With these considerations in mind, we decided to test the hypothesis that, similar to methotrexate, therapeutic cell delivery to the brain parenchyma requires blood-brain barrier disruption (BBBD). Since BBBD sporadically but reproducibly leads to transient focal motor seizures in patients [7,10] and animals [10,16] we also wished to assess whether BBBD paired to cell injection bears additional side effects. To ensure an immediate translation to human studies, we used a large animal model of BBBD and tools that are commonly employed in the clinical setting. To exaggerate the possible morbidity of BBBD + cell therapy, we used the cytotoxic human-derived TALL-104 cells in slightly immunocompromised pigs.

## Methods

### Animal handling and anesthesia

Experimental research was performed according to the International guidelines and the Cleveland Clinic IACUC office. Experiments performed are included in approved IACUC protocol. A total of eight 35 kg Yorkshire pigs were placed under anesthesia using isofluorane (1-1.5%). Anesthesia was induced by intramuscular ketamine with the addition of xylazine (Rompun; 1-2 mg/kg) and atropine (0.02 mg/kg). Pigs were immunosuppressed with Cyclosporine-A to prevent possible immunological reactions in response to the injection of human-derived cells.

### Blood-brain barrier disruption procedure

Under general anesthesia, pigs underwent MRI (coronal, longitudinal T1 and perfusion) prior the hemispheric BBB disruption procedures. Immediately after, pigs were transferred to the operating room. In the supine position, the femoral artery of the pig was cannulated and, under fluoroscopic guidance, the internal carotid artery catheterized. We performed angiography with a right femoral artery cannulation and found that the swine cerebral circulatory system demonstrated a plexus of very small vessels (*rete mirabile)*. The base of the brain is perfused by the ascending pharyngeal artery and reconstitutes downstream into the internal carotid artery. Angiography was also performed to determine the rate and volume of an injection to opacity and possible cross-filling of the contralateral (control) side. Mannitol (25%) was then administered intra-arterially via the catheter at a predetermined rate of 1-4 cc/sec for 30 seconds, ranging from ineffective dosages (30 ml/pig) to effective 90-120 ml/pig [7-10]. Procedures conducted using low mannitol dosage or the contralateral hemisphere in the effective BBBD procedures were used as control.

### Gd^++ ^-TALL-104 cells injection

Gd^++^-loaded TALL-104 cells were provided by AbiogenPharma S.p.a. in a sterile formulation ready for injection. TALL-104 cells are of human origin and quality controlled for the following: mycoplasma contamination, bacterial contamination, fungal contamination, viral contamination, antigenic profile, cytotoxic profile, genotype and purity [17,18]. A small sample of cells was stained with Trypan Blue to check the viability of the cells. Immediately before each experiment, a 20 ml syringe filled with Gd^++^-TALL-104 was scanned by MRI to confirm the presence of intracellular contrast. Intra-arterial TALL-104 cells were infused immediately after BBBD (7-8 × 10^9 ^in 20 ml of sterile saline) The number of cells was chosen based on previous studies conducted in rodents and ongoing clinical trials for the treatment of systemic tumors [19-22]. Electroencephalography (EEG) and trans-cranial Doppler (TCD) were performed during each procedure. A second MRI was performed after the BBBD procedures to evaluate the distribution of Gd^++^-TALL-104 in the brain.

### Electroencephalography

The following equipment was utilized to collect EEG data during the procedures: Nihon Kohden EEG System (Nihon Kohden JAPAN) Nihon Kohden Neurofax EEG 9000 Version 5-72 running on a WIndows XP platform using DELL Optiplex GL280. A non-cephalic reference was utilized for comparison. Tracings were collected on a Nihon Kohden JE910-A jackbox, using sterile stainless steel subdermal needle electrodes (13 × 0.40 mm with 0.5 × 27 Gauge) manufactured by Axon Systems Hauppage NY. The system was electrically isolated from the pig and the equipment used in performing angiographic procedures. Diadem (National Instrument, USA) was used for joint-time frequency analysis of electrical signals.

### Trans-cranial Doppler

PMD150transcranial Doppler (Spencer Technologies, Seattle, WA) was used to monitor cerebral blood flow using power M-mode Doppler (PMD). PMD is a format for presenting Doppler data which is in the style of traditional motion mode imaging (depth from the probe on the vertical axis and time on the horizontal axis), but shows power of the reflected Doppler signature over depth instead of simple gray scale tissue reflection amplitude. TCD probes were fixed on the head of the pig on the orbital and in the temporal bones.

### Histology and Immunohistochemistry

At the end of the BBBD procedure, five pigs were perfused with Evans Blue (2%, 4 ml/Kg) to allow histological evaluationn of BBBD. At the end of each experiment the brain was removed from the skull and formalin fixed. Parenchymal extravasate of Evans Blue was first assessed by visual inspection. Histological and immunohistochemical (IHC) staining were then performed from blocks of parietal and temporal regions correspondent to the territory supplied by the internal carotid artery. For histological studies, sections (30-35 μm) stained with 1% Cresyl violet (CV) for cytoarchitectural analysis and presence of gross cellular extravasates in correspondence of vessels. Free floating sections were stained with anti-human CD8 to detect human TALL-104. We used: polyclonal anti-human CD8 (Ab 4055, 1:100, Abcam); monoclonal anti-GFAP (G 3893; 1:100, Sigma, Saint Louis, Missouri, USA); NEUN (MAB 377, 1:500, Chemicon) Secondary antibodies: Texas red affinipure donkey anti-mouse IgG (1:100 Jackson Laboratories Inc., West Grove, PA, USA), and Fluorescein isothiocyanate (FITC)-conjugated affinipure donkey anti-rabbit IgG (1:100, Jackson Laboratories Inc., West Grove, PA, USA). Autofluorescence was blocked with Sudan black B. Sections were analyzed by confocal microscopy.

### Cell quantification

Twenty seven slices from each brain hemisphere (temporal lobe) were used to quantify cellular extravasation. Pictures were taken using a bright field microscope (400 × 600 pixels/inch). Pictures were imported into Adobe Photoshop 7.0 and transformed into a grayscale image. Images were then processed using Phoretix 2D for cell quantification (spot detection). Sensitivity and size of signal was kept constant and controlled throughout out the analysis.

### Statistical analysis

We used Origin 7.0 (Origin Lab, Northampton, MA, USA) and Jump 7.0 (SAS) software. Shapiro-Wilk test was used to evaluate the normal distribution of the data. Data are indicated as mean ± SEM. Student t-test was used for direct comparison of two populations of data. p < 0.05 was considered statistically significant.

## Results

**Figure 1:**
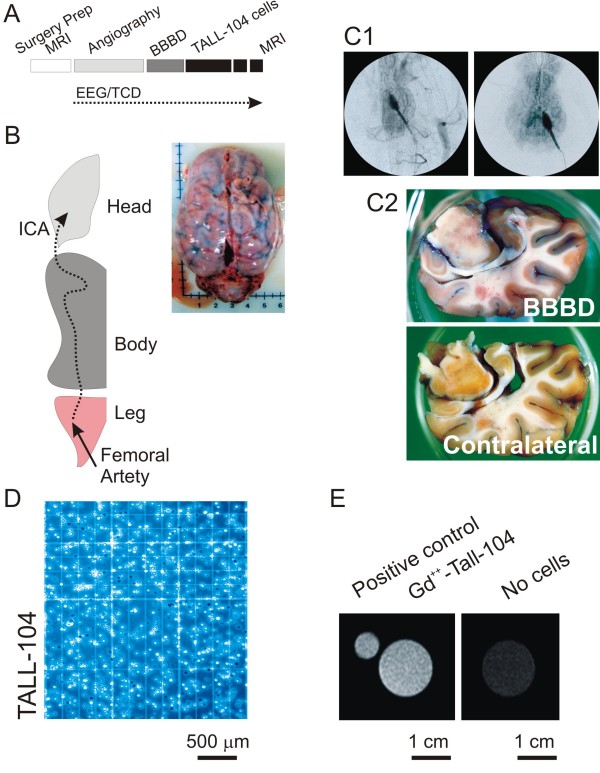
**Schematic representation of the surgical procedure and quality control of Gd^++^-TALL-104 cells**. **A) **Experimental steps common to all the experiments. BBB disruption (BBBD) was performed after baseline MRI. Continuous electroencephalography (EEG) and trans-cranial Doppler (TCD) recordings were performed. MRI scans were performed before and 48 hours after the procedures. **B) **Catheter was placed in the femoral artery and angiographically guided to the ICA at the base of the brain. **C1) **Localization of the tip of the catheter and angiographic exploration. Note the extent of contrast diffusion to the hemisphere correspondent to the ICA cannulated and a modest spread to the contralateral side. Note also the venous return after contrast injection. **C2) **Successful BBBD lead to hemispheric leakage of Evan's Blue in the brain parenchyma. Note the negligible amount of blue signal in the contralateral side. **D) **Cell vitality was demonstrated by lack of Trypan Blue penetration. **E) **MRI scans (T1) confirmed that TALL-104 cells retain Gd^++^.

For the experiments described herein, we used a total of 8 pigs. Animals underwent intracarotid (ICA) injection of Gd^++^-TALL-104 after BBBD (Figure 1A-B). After a preliminary pre-operative MRI scan and following anesthesia induction, we first tested the localization of the tip of the catheter (Figure 1 C1) by iodinated contrast injection. Angiography was also used to determine the extent of contralateral diffusion of contrast, as well as to visualize the extent of venous loading. Hemispheric opening of the BBB was confirmed by Evan's Blue staining (Figure 1 C2). Immediately prior to the actual injection, cells were prepared under sterile conditions and counted. Cells were tested for vitality (Trypan Blue, Figure 1D) and capacity to retain the radiological marker Gd^++ ^(Figure 1E).

Hyperosmotic mannitol was injected into the ICA as described in the Methods section. Immediately after Gd^++^-TALL-104 cells were injected (velocity = 1 ml/sec) using the same catheter. Throughout the procedures, we monitored cerebral blood flow, EEG activity and vital signs.

**Figure 2:**
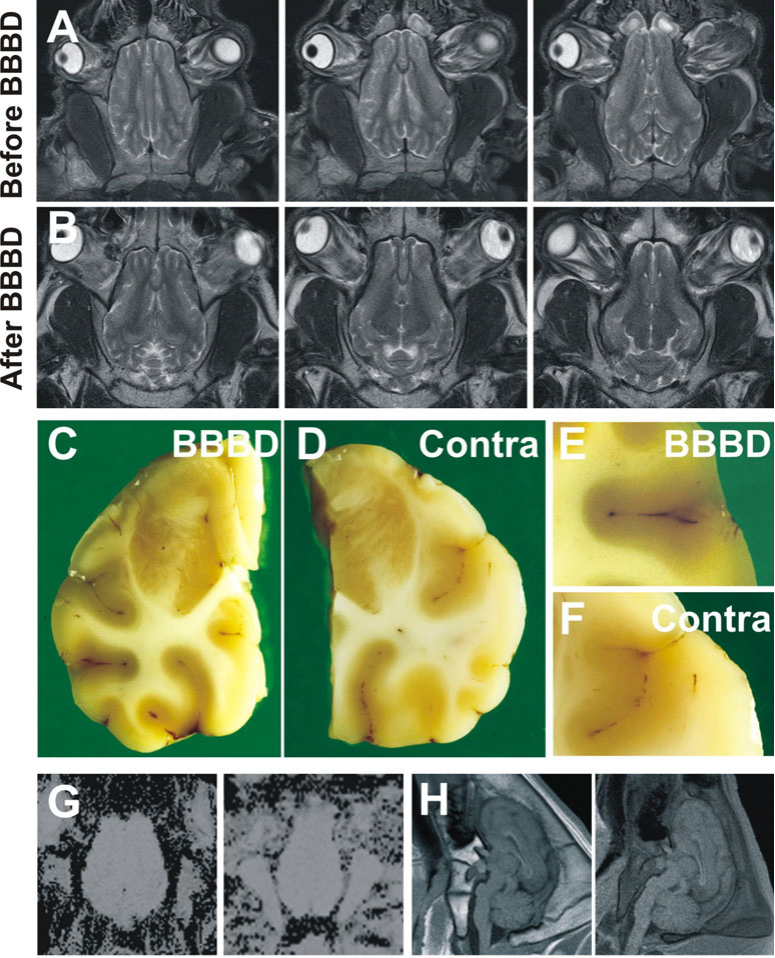
**Lack of BBB opening can be detected radiologically or histologically**. **A-B) **Example of an ineffective BBBD procedure. A low ineffective concentration of mannitol was used as control (*non*-BBBD, see methods). Radiological (MRI, T1) scans were similar before and after the BBBD procedure. Note the lack of Gd^++ ^signal in the scans taken after BBBD procedure, suggesting lack of cellular brain penetration. **C-F) ***Ex-vivo*, the absence of Evans Blue brain leakage further confirmed the lack of BBB opening in this animal. BBBD and contralateral sides were virtually identical. **G-H) **Perfusion scans and sagittal T1 sequences were taken after BBBD procedures to rule out the presence of gross anatomical alteration associated with the procedure.

We used several independent methods to evaluate the toxicity of the cell injection procedure. At the concentration used (see methods, [19-22]), TALL-104 were well tolerated. Figure 2A-B shows examples of MRI scans and gross and anatomic observations in animals injected with a low, ineffective dosage of mannitol (1 ml/sec, 30 seconds) followed by Gd^++^-TALL104. Note that no discernable MRI changes were seen. MRI appearances of pre-procedure and post-procedure scans were identical. This was further confirmed by gross anatomic observations of Evans Blue extravasation (Figure 2 C-F). We tested the contralateral brain by histological and gross anatomical means. In all cases, the two hemispheres were found to be virtually identical (Figure 2 C-F). Another functional parameter that was assessed following the procedure was perfusion-weighed imaging to unveil possible infractions or altered brain hemodynamics after the procedure. These perfusion maps were then analyzed and compared to normal (pre-BBBD) imaging as shown in Figure 2G. We never observed any change in perfusion patterns in the animals tested. Sagittal MRI scans were also performed to rule brain or cervical cord damage (Figure 2H).

**Figure 3:**
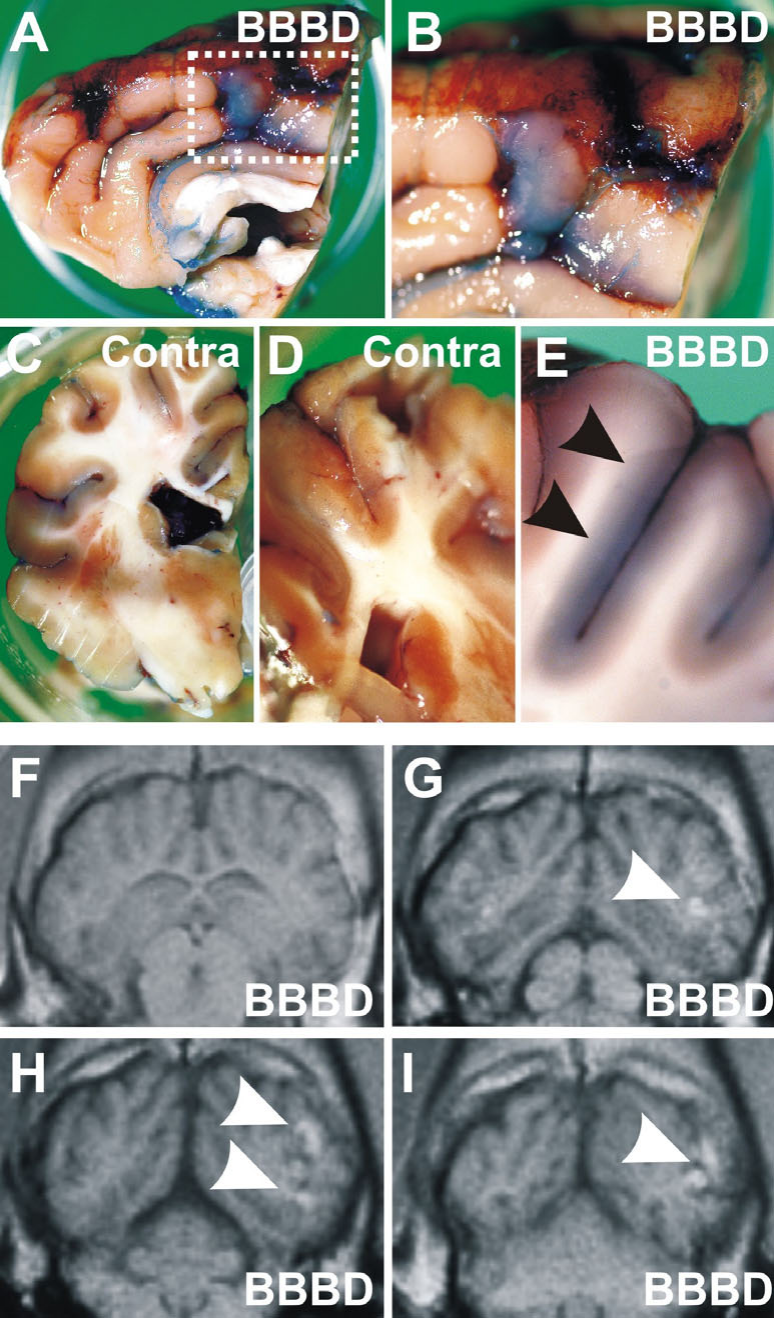
**Evaluation of a successful blood-brain barrier disruption**. **A-B) **Note Evans Blue leakages in the parietal BBBD hemisphere compared to the correspondent contralateral areas **(C-D). B and E **show additional details of parenchymal dye extravasations (*arrowheads*). **(F-I) **Histological findings were confirmed by MRI (T1). Serial images show Gd^++^- positive signals in the same regions where Evans Blue leakage was detected (*arrowheads*).

In 1/3 of the procedures an excellent opening of the BBB was achieved when using mannitol 4 ml/sec, 30 seconds, as judged by the extravasation of Evans Blue and comparison with the contralateral hemisphere (Figure 3A-E). In these procedures, the extent of leakage was comparable to what was previously reported [10]. In particular, we confirmed that in the BBBD hemisphere a faint overall leakage pattern was punctuated by spots of more pronounced dye extravasation (Figure 3B-E). These spots were comparable to the pre-sacrifice contrast-enhanced MRI scans in pigs injected with Gd^++ ^loaded cells immediately after mannitol. Note that the approximate size and location of extravasations measured by Evan Blue were similar to what was seen *in vivo *(Figure 3G-I). Note also the lack of extravasation and Gd^++ ^signal in the contralateral side. In the remaining 2/3 of procedures, the extent of Evans blue extravasation was limited to a few sites of extravasation and was similar to control procedures shown in Figure 2. The results so far presented confirmed that BBBD does not always result in maximal "opening" of the BBB and that successful openings are characterized by a patchy distribution of leakage spots [10].

**Figure 4:**
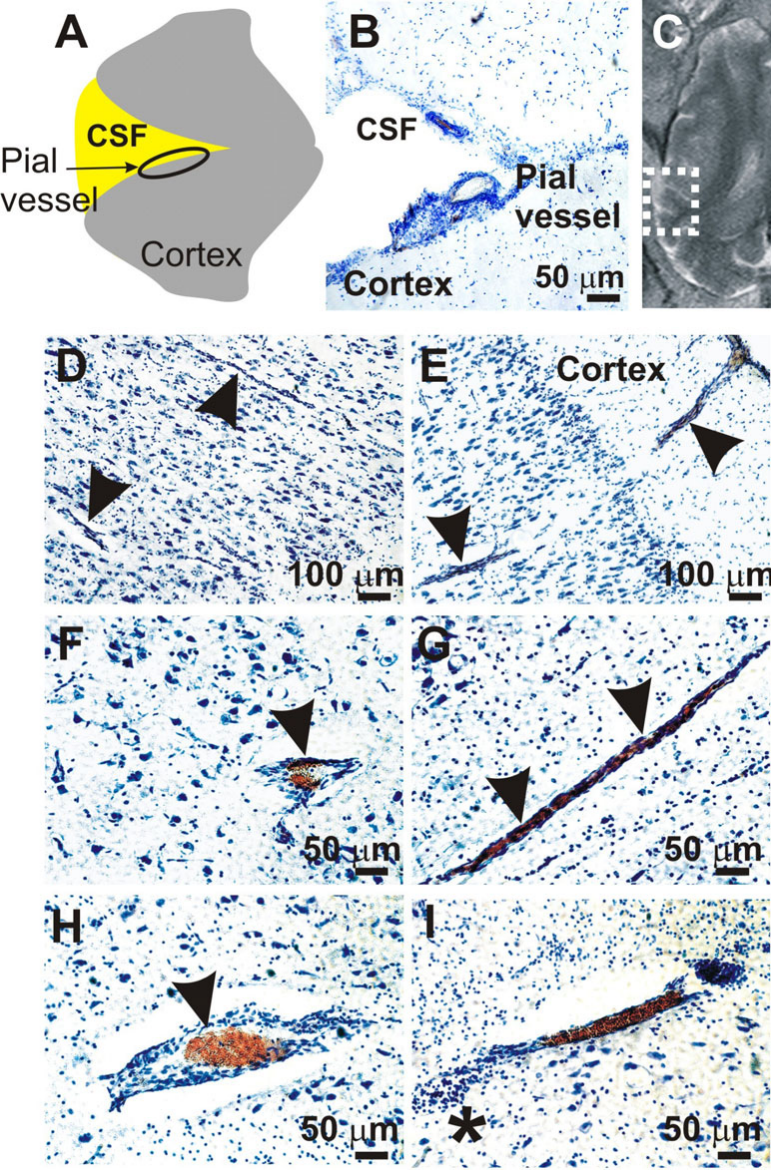
**Lack of microscopic evidence of cell brain extravasations after modest BBBD procedures**. **A-C) **When BBB disruption was not induced (see also Figure 2), cellular extravasation (as detected by Cresyl Violet) was limited to the Virchow-Robin space or to the proximity of superficial meninges. Modest and localized Gd^++^extravasation was observed in the same regions. **D-H) **Cresyl Violet staining showed that parenchymal vessels were generally devoid of cellular extravasates. The reddish stain is indicative of hemoglobin accumulations in erythrocytes. *Arrowhead *indicates vessel in both BBBD and contralateral hemispheres. **I) **Small clusters of cells were infrequently observed in the proximity of vessels (*asterisk*).

Brains were analyzed microscopically and the extent of cellular extravasation quantified by traditional histological means (Cresyl Violet) or immunostaining (human anti-CD8). Figure 4 shows an example of histological appearance of brain sections from an animal where BBB disruption was not induced; this was also previously quantified by contrast-enhanced MRI and *post-mortem *histological evaluation of Evan Blue (Figure 2). Note that the MRI test was performed 24 hours after BBBD while the histology was done 48 hours after the procedure. Under conditions of absent BBB disruption, cell extravasation was confined to the Virchow-Robin space surrounding large penetrating pial vessels or in proximity of superficial meninges (*arrow head *in Figure 3D-H) while isolated, small clusters of cells were infrequently observed (*asterisk *in Figure 2I).

**Figure 5:**
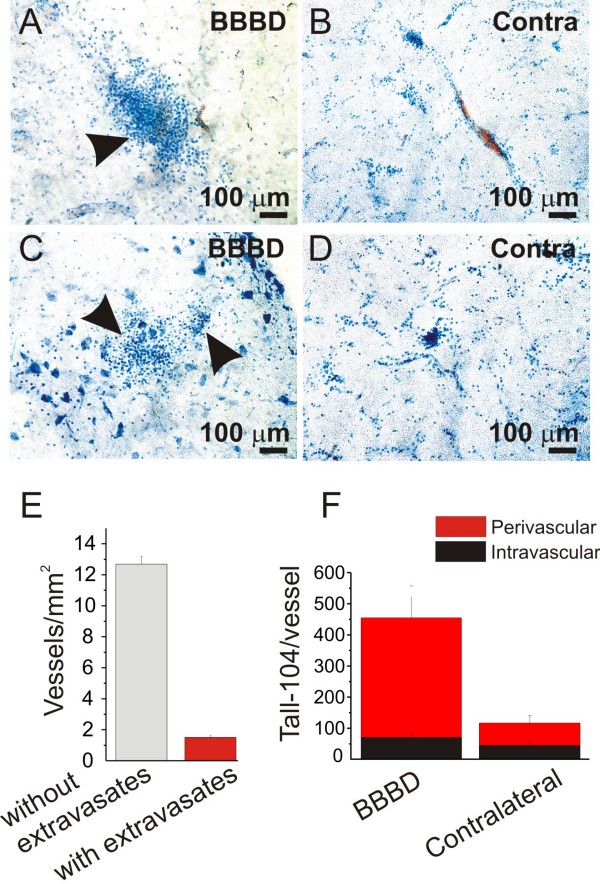
**Pattern of cell brain extravasations after successful BBBD**. **A, C) **Accumulation of cells in the proximity of vessels was observed in the BBBD hemisphere. Groups of cells were detected in the sections analyzed (See Methods for Details). **B, D) **Conversely, fewer cells were detected in the contralateral non-BBBD hemisphere. **E-F) **A significant number of vessels was associated with cellular extravasates. These vessels were mostly confined to the BBBD hemisphere (F). Cell extravasates were distributed in the immediate proximity of the disrupted vessels (perivascular compartment, *red bar *in F), while negligible amounts were measured in the parenchyma (*not shown*).

These measurements were repeated in pigs where a successful BBB opening was achieved (Figure 5). Several spots of cellular extravasates were observed in association with pial and parenchymal vessels in the BBBD hemisphere (Figure 5A, C) Figure 5E shows the quantification of the number of vessels associated with cellular extravasates. Note that the contralateral hemisphere is used as internal control for the BBBD side (Figure 5B, D). Thus, little amount of cells was observed perivascularly in the contralateral side (Figure 5, panel F). These data were confirmed both radiologically (Figure 3) and histologically (Figure 5). In spite of the fact that extravasation was obvious in these animals both radiologically and histologically, most of the cells were confined to the perivascular space and only a small percentage of cells was found in the brain parenchyma (Figure 5F)

**Figure 6:**
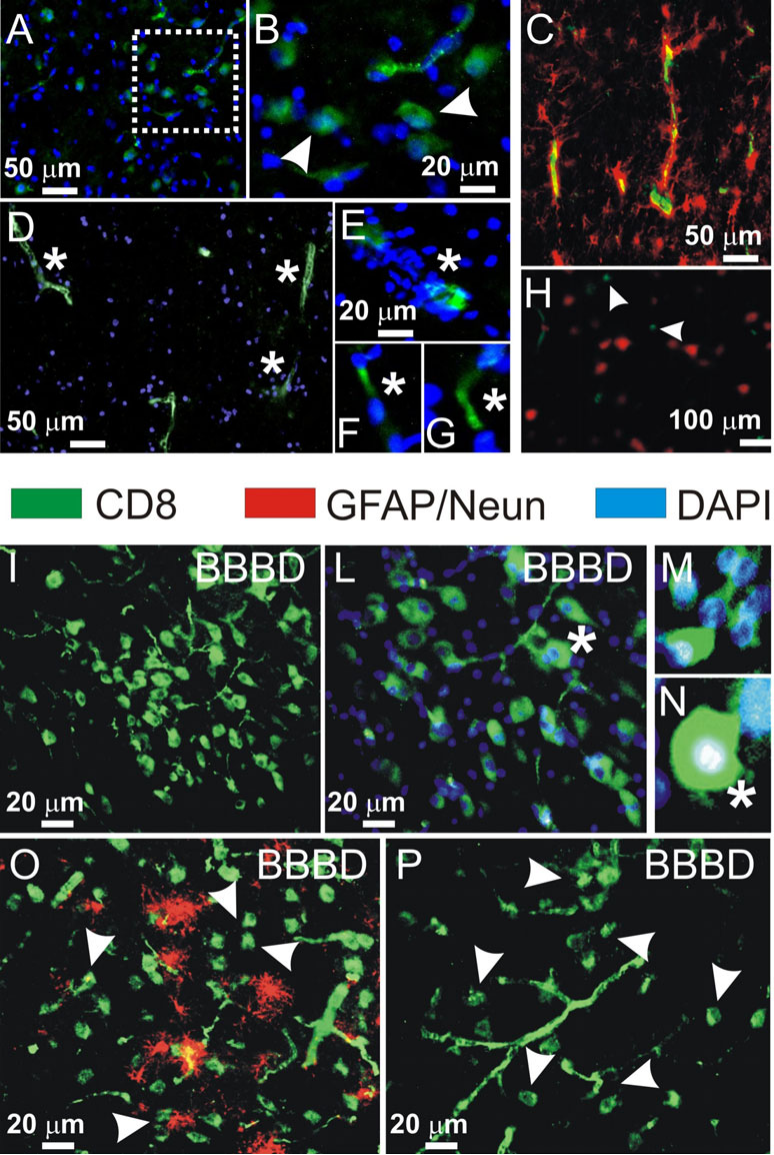
**Immunohistochemical characterization of the cellular extravasates**. Anti-human CD8 antibody was used to identify TALL-104 cells within the cellular extravasates. **A-D) **Negligible amounts of CD8 positive cells were detected in the perivascular space when BBB was not breached (see also Figures 2 and 4). *Arrowheads *in B and H show examples of the rare extravasated cells. **(E-G) **Note that CD8 immunoreactivity was mostly confined in the intravascular space. **I-P) **Brain sections obtained from successful BBBD procedures display elevated anti-CD8 immunoreactivity in correspondence of perivascular aggregates of cells (*arrowheads*). See also Figure 3 and 5.

We performed immunocytochemical analysis to detect human CD8-positive cells to unequivocally determine the cell type in extravasates (TALL-104*vs*. autologous white blood cells). The evaluation was conducted in animals where the BBB was breached successfully (Figure 6I-P) as well as in those where BBBD was not induced (Figure 6 A-H). Cell extravasates seen histologically comprised invariably of CD8^+ ^cells. Lack of BBB opening resulted in negligible number of CD8^+ ^cells in the perivascular space (Figure 6A, C-D). CD8^+ ^cells were confined within the vascular bed (Figure 6C, E-G). After successful BBB opening, several cellular aggregates were found in correspondence of vessels (Figure 6I-P).

**Figure 7:**
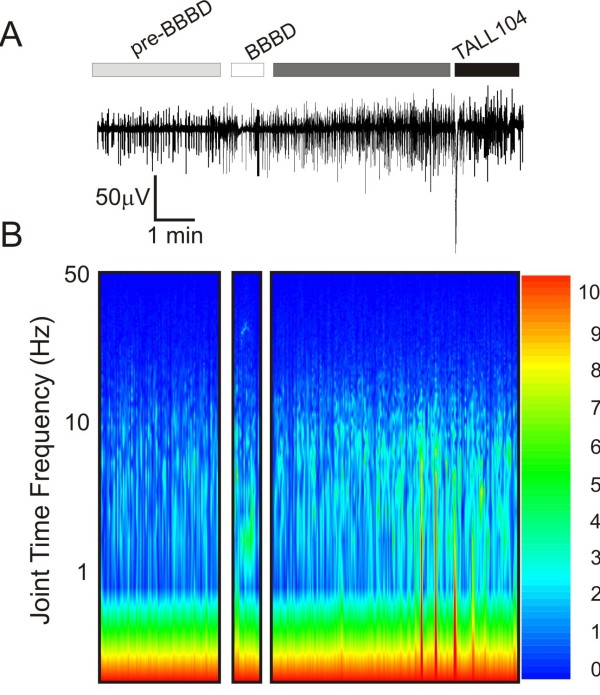
**EEG monitoring during BBBD and cell injection**. **A) **EEG recordings revealed abnormal neuronal activity after a successful BBBD. However, no frank seizures were observed in these animals. The injection of cells did not exacerbate the EEG alterations provoked by the BBBD procedure. **B) **A joint time FFT frequency plot reveals the changes in EEG frequency over time and in relationship to BBBD. Note the increase in 5-10 Hz frequencies after mannitol injections and the increase in spike amplitude (color coded).

**Figure 8:**
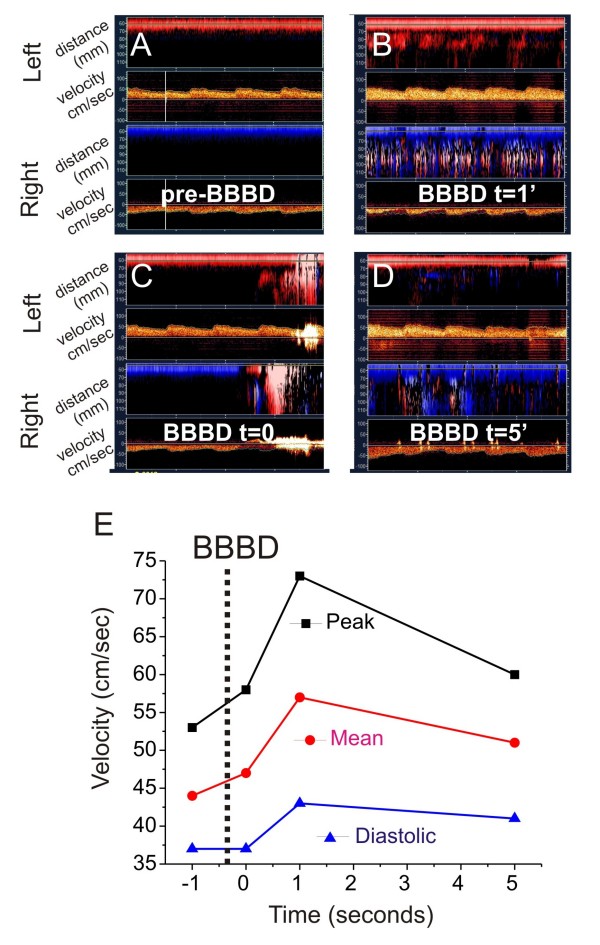
**Transcranial Doppler monitoring during BBBD and cell injection**. We confirmed the safety of the BBBD + cell injections. **A-D**) Cerebral blood flow velocity was slightly increased immediately after BBBD (See Text for Details).

The neurophysiological effects of BBBD and cell injection on cerebral function were measured by EEG (Figure 7). A joint time FFT frequency plot was constructed to demonstrate even small changes in EEG frequency occurring during of after BBBD. In all experiments, mannitol injection caused a transient "loss" of EEG signal, presumably due to the rapid passage of high osmolarity blood through the region of recording[10]. This electrical silence lasted a few seconds to one minute and was also reflected by the change in TCD signal (Figure 8). In general, EEG recordings were unremarkable up to one hour post-BBBD, at which point the animal was weaned off respirator and anesthesia. No frank behavioral or electrographic seizures were observed in the animals tested. When maximal BBB disruption was achieved, changes in EEG frequency were observed (Figure 7). These changes consisted of increased signal intensity in the 5-10 Hz range (Figure 7B).

While steady-state (48 hrs) post-operative changes in CBF were monitored by perfusion MRI, the acute cerebrovascular safety of the procedure was confirmed by transcranial Doppler monitoring (Figure 8). Cerebral blood flow velocity was slightly increased in the immediate period after blood-brain barrier disruption (Figure 8E). Within 10 minutes after the procedure, CBF values returned to pre-BBBD levels. Note that mannitol injection caused a significant perturbation of the signal, presumably due to the altered acoustical properties of hyperosmotic solutions. This change did not, however, interfere with measurements of Doppler velocity, but temporally corresponded to the apparent loss of EEG signal.

## Discussion

To our knowledge, this is the first demonstration that MRI-detectable cytotoxic cells with proven anti-neoplastic activity can transverse the BBB by clinically realistic means. Our data show that targeted CNS cell therapy requires BBB disruption. The virtual absence of toxicity, the high anti-tumor activity of TALL-104, and the clinical feasibility of human osmotic BBBD suggest that this approach may be used to treat brain or spinal cord tumors. In addition, BBBD may favor CNS entry of other cells that normally lack CNS tropism.

### Cell trafficking at the BBB

It is generally assumed that trans-BBB trafficking of white blood cells is a common and frequent occurrence (for a critical discussion see [5]). Our results in contrast propose that trans-BBB penetration of cells is a relatively infrequent event and require BBB opening. The results showed that even when the BBB is breached, cell extravasation in the brain parenchyma is not widespread but mostly confined to the perivascular space. No significant cell entry was observed in non-BBBD procedures (Figures 2 and 4) or in the contralateral side of BBBD procedures (Figures 3 and 5).

It is, however, well accepted that antigen plays a major role in trafficking of cells to the brain. Non specific CD8 T cells which has not been activated through their T cell receptor will not cross the BBB, while their counterparts where antigen presentation and subsequent activation has occurred, will. We may therefore conclude that in our experiments TALL-104 cells were not capable of greatly entering the CNS because of lack of prior antigen activation. This also implies that the presence of tumor cells in the brain could greatly increase cell delivery. Cell trafficking in brain with an intact or only slightly breached BBB, was limited to the subdural regions. Trials with tumor-bearing animals will unveil whether these levels of extravasated cells after BBBD are sufficient to exert toxic activity against brain neoplasms.

There are several issues that may have confounded the interpretation of our results. The possibility exists that exposure of BBB endothelial cells to hyperosmotic media is not sufficient to disrupt tight junctions. This is a reasonable concern, given the fact that we and others have shown that mannitol-induced BBBD causes variable extent of "opening" [7,10]. This, however, was a controlled variable in our study, since we measured pronounced extravasation of Evans Blue (and FITC-labeled albumin [10]). When the extent of clinical or experimental BBBD was measured radiologically, a full range of efficacy was shown, from nil to excellent [7,10]; in addition, the clinical response to chemotherapy was highly suggestive of improved drug delivery of methotrexate to the brain. Thus, it appears that leakage of small molecules (*e.g.*, dyes) or dye-bound protein does not immediately translate into cell extravasation in the parenchyma. This was indirectly confirmed by numerous studies where extravasation of albumin, immunoglobulins or other protein was observed in brain regions with little cell extravasation beyond the perivascular space [23-27].

### Clinical significance and translation

The safety of the combined approach used (cytotoxic cells and blood-brain barrier disruption) suggests that this is a viable means for targeted cell delivery to the brain. Our previous experiments have shown that successful BBBD promote seizures [10]. Based on this we cannot therefore speculate on whether or not T cell entry is necessary for seizure induction. However, the delay between injection of mannitol, BBB disruption and seizure generation (few minutes) argues against a sophisticated cell-to-cell interaction or immunological release of antibodies. Further experiments are necessary to clarify the role, if any, of specific cell sub-types in the generational seizures. It does remain, however, possible, that epilepsy but not seizures are maintained by WBCs which enter the brain. These investigations were beyond the scope of our study but clearly bare clinical significance.

Our previous experiments have shown that in patients BBBD is variable even when the surgical team remains the same and the protocols are respected [7,10]. However, in these human trials as well as in the experiment presented here we did not use sham - operated animals. This is due in part to the fact that large animal experiments are extremely complicated and approval for sham procedures are difficult to obtain; however, there is also scientific supports that make these experiments unnecessary. In fact, we and others have shown that BBBD by intracarotid injection does not affect the contralateral side. In other words, the contralateral brain acts as a control [7,10]. The cell extravasation data shown in Figure 5 show a preference for the disrupted hemisphere thus confirming that BBBD allows better extravasation of the cells. This is further supported by the fact that seizures induced by BBBD invariably originate in the disrupted hemisphere and have motor component spread to the contralateral side of the body [10].

Our experiments may show promise for the treatment of primary or metastatic brain tumors. *In vitro*, TALL-104 cells show significant cytotoxic activity against human glioblastoma cell lines (U-87 MG, U-251 MG, and A1690), the medulloblastoma cell lines DAOY, D283 Med, and D341 and the epidermoid cancer cell line A431. TALL-104 cells also cause lysis of human-derived brain tumor cells, sparing normal brain cells [17,18]. To date, only one report showed the safety and the potential therapeutic effects of TALL-104 cells in reducing brain tumor burden [17,18]. The authors investigated the efficacy of TALL-104 cells in reducing a human A431 carcinoma implanted in brain. However, TALL-104 cells were directly administered into the brain (intraventricularly). Of relevance is the fact that no adverse reactions (*e.g.*, allergic encephalitic) were triggered by TALL-104 brain injection [17,18]. Interestingly, in rodents, TALL-104 cells did not penetrate the brain when injected intravenously and without manipulation of the BBB [28]. TALL-104 cells were well tolerated by patients enrolled in two phase I clinical trial for systemic cancer at the doses and regimen tested [22]. There are numerous other cell lines that may be used for cellular therapy of brain tumors, and the example shown here simply addressed the feasibility and safety of this approach in an animal model that closely mimics the clinical reality.

## Conclusions

Our data show that brain penetration of peripherally circulating cells requires BBB disruption and it is limited to the immediate perivascular space. Specifically, MRI-detectable cells can be forced to transverse the BBB using clinically realistic means.

## Authors' contributions

NM: carried out surgeries, brain dissection, interpretation of data and manuscript draft; QT, LF, ND and PR: carried out animal surgery and immunohistochemistry; MN: carried out immunohistochemistry and relative quantification; TM: carried out some of the animal surgery; ST: provided Tall-104 cells; DJ: conceived of the study, participated in its design and coordination All authors read and approved the final manuscript
